# Robotic versus laparoscopic heller myotomy for esophageal achalasia: an updated systematic review and meta-analysis

**DOI:** 10.1007/s00423-025-03648-1

**Published:** 2025-02-17

**Authors:** Alberto Aiolfi, Riccardo Damiani, Michele Manara, Francesco Cammarata, Gianluca Bonitta, Antonio Biondi, Davide Bona, Luigi Bonavina

**Affiliations:** 1 Division of General Surgery, Department of Biomedical Science for Health, I.R.C.C.S. Ospedale Galeazzi– Sant’Ambrogio, University of Milan, Via C. Belgioioso, 173, 20157 Milan, Italy; 2 Department of General Surgery and Medical Surgical Specialties, Surgical Division, G. Rodolico Hospital, University of Catania, Catania, 95131 Italy; 3 Department of Biomedical Sciences for Health, Division of General and Foregut Surgery, University of Milan, IRCCS Policlinico San Donato, Milan, Italy

**Keywords:** Esophageal achalasia, Dysphagia, Myotomy, Laparoscopy, Robotic, Esophageal perforation

## Abstract

**Background:**

The surgical treatment for esophageal achalasia has evolved over the years, with laparoscopic Heller myotomy (LHM) and partial fundoplication becoming widely used worldwide. More recently, an increased interest in the robotic Heller myotomy (RHM) has arisen.

**Purpose:**

Compare short-term and functional outcomes of RHM vs. LHM.

**Methods:**

Systematic review and meta-analysis. PubMed, MEDLINE, Scopus, Web of Science, Cochrane Central Library, and ClinicalTrials.gov were queried. Primary outcome was esophageal perforation (EP). Risk ratio (RR), standardized mean difference (SMD), and 95% confidence intervals (95% CI) were effect size and relative inference measures. PROSPERO Registration Number: CRD42024512644.

**Results:**

Fourteen observational studies (12962 patients) were included. Of those, 2503 (19.3%) underwent RHM. The patient age ranged from 34 to 66 years and 51.7% were males. EP occurred in 259 patients (1.99%). The cumulative incidence of EP was 1.67% for RHM and 2.07% for LHM. Compared to LHM, RHM was associated with a reduced risk of EP (RR: 0.31; 95% CI 0.16–0.59). No differences were found in term of dysphagia requiring reoperation or additional endoscopic procedures (RR: 0.47; 95% CI 0.20–1.09) and postoperative Eckardt score (SMD: -0.42; 95% CI -0.94, 0.11). Blood loss, conversion to open, operative time, and hospital length of stay were comparable.

**Conclusions:**

RHM may be associated with a reduced risk of EP compared to LHM. However, because of selection bias, diverse surgeon expertise, variations in surgical technique, and prior endoscopic procedures these findings should not be viewed as conclusive while the superiority of one approach over the other remains to be established.

**Supplementary Information:**

The online version contains supplementary material available at 10.1007/s00423-025-03648-1.

## Introduction

Achalasia is a rare primary disorder of esophageal motility characterized by the inability of the lower esophageal sphincter (LES) to relax and absence of peristalsis in the esophageal body [1–3]. As the condition progresses, patients commonly experience dysphagia along with chest pain, food regurgitation, airway aspiration, and malnutrition [4, 5]. Three distinct manometric subtypes of achalasia have been described by the Chicago International Study Group, with different prognosis in terms of symptom resolution after surgical or endoscopic therapy [6, 7].

The treatment approaches for achalasia have evolved over the years, with laparoscopic Heller myotomy (LHM) and partial fundoplication becoming the most widely used surgical technique globally [8–10]. More recently, due to the better 3-D visualization, instrument stability, and superior dexterity provided by the robotic platforms, there has been an increased interest in the robotic Heller myotomy (RHM) [11–16]. It has been hypothesized that RHM may offer better clinical outcomes compared to LHM, particularly in terms of reduced postoperative pain, shorter hospital length of stay (HLOS), and decreased risk of intraoperative mucosal esophageal perforation (EP) [14, 17–23]. While few studies and meta-analyses have been published, conclusive evidence supporting the superiority of one treatment over the other is still unavailable [14, 18, 24, 25]. 

Hence, this updated systematic review and meta-analysis aims to compare outcomes of RHM vs. LHM for the treatment of esophageal achalasia in term of short-term and functional outcomes.

## Materials and methods

This study was conducted following the Preferred Reporting Items for Systematic Reviews and Meta-Analyses (PRISMA) statement and Meta-analyses Of Observational Studies in Epidemiology (MOOSE) guidelines [26, 27]. Institutional Review Board approval was not required. Multiple databases, including PubMed, Scopus, MEDLINE, Web of Science, ClinicalTrials.gov, Cochrane Central Library, and Google Scholar, were utilized for the search process. A combination of the following MeSH (Medical Subject Headings) terms was utilized for the literature search: “robotic,” “robot*,” “laparoscopic,” “laparosc*”, “Heller myotomy,” “myotomy”, “achalasia” AND “esophageal achalasia”. The complete search strategy is described in Appendix A. The last date of the search was 1st July 2024. Two authors (RD, AA) reviewed the title of each study and extracted relevant abstracts. The search was further enhanced by examining the references of each article. The study protocol has been registered with the international prospective register of systematic reviews (PROSPERO registration number: CRD42024618855).

### Eligibility criteria

The criteria for inclusion were (a) clinical studies reporting a comparison between RHM and LHM in adult patients (> 17 years) diagnosed with esophageal achalasia related symptoms; (b) English written; (c) in cases where multiple studies were based on the same dataset, the study with the longest follow-up period or the largest sample size was selected, and (d) for duplicate studies, only the study with the most complete dataset was included for quantitative analysis. The exclusion criteria were (a) non-English articles; (b) studies with a lack of clear outcome comparison for LHM vs. RHM; (c) studies not reporting the pre-defined primary outcome; (d) case series and case reports.

### Data extraction

The following variables were collected: the authors, year of publication, country, study design, the number of patients, age, sex, body mass index (BMI), American Society of Anesthesiologists (ASA) score, type of achalasia according to the Chicago classification, previous endoscopic treatment, myotomy length (cm), technical details repairing, the method for myotomy, type of fundoplication, short-term outcomes, functional outcomes, and need for redo surgery of endoscopic dilation. Three investigators (RD, AA, MM) independently gathered and analyzed the data, which were compared at the conclusion of the review. Discrepancies were resolved through discussion with a senior author (LB).

### Outcomes

Primary outcome was EP. When specified, the timing of EP diagnosis was defined as intraoperative or postoperative. Secondary outcomes were operative time (minutes), estimated intraoperative blood loss (ml), hospital length of stay (days), post-operative Eckardt score, conversion to open, postoperative dysphagia requiring reoperation or endoscopic procedures.

### Quality assessment

Two authors (RD, AA) performed separate and independent evaluations of the methodological quality of each study. The ROBINS-I tool was used, taking into account confounding bias, selection bias, classification bias, intervention bias, missing data bias, outcome measurement bias, and the bias in study reporting [28]. Each domain was rated as “yes”, “probably yes”, “probably no”, or “no”, leading to overall risk judgments categorized as a low, moderate, serious, or critical bias risk. The quality of the overall evidence from the studies was evaluated using the Grading of Recommendations, Assessment, Development, and Evaluation (GRADE) tool (https://www.gradepro.org; accessed on 15th September 2024) [29, 30]. 

### Statistical analysis

The findings of this study were synthesized using frequentist random-effect meta-analysis, aggregating risk ratios (RR). The DerSimonian–Laird estimator was employed to determine the variance of the true effect size (τ2), alongside an inverse-variance method [31, 32]. Heterogeneity across studies was assessed through the I [2] index and Cochran’s Q test [33]. Levels of statistical heterogeneity were categorized as low, moderate, and high for I [2] values of 25, 50, and 75%, respectively, with significance set at *p* < 0.10. [34, 35] Confidence intervals (CI) at 95% were computed using the Wald-type method for pooled measurements, while the Clopper-Pearson method was applied for individual intervals. The 95% CI for the I [2] index followed Higgins and Thompson’s approach [36]. Prediction intervals for the treatment effect of new studies were calculated based on Borenstein et al. [33]. Sensitivity analysis was conducted by iteratively excluding one study at a time to ensure the robustness of the overall results, considering varying sample sizes across studies. Statistical significance was defined as a two-sided *p* value of less than 0.05. All analyses and visualizations were performed using R software version 3.2.2 [37]. Consistent with Cochrane guidelines, publication bias assessment was not conducted for outcomes including less than 10 studies per data comparison.

## Results

### Systematic review

**Fig. 1:**
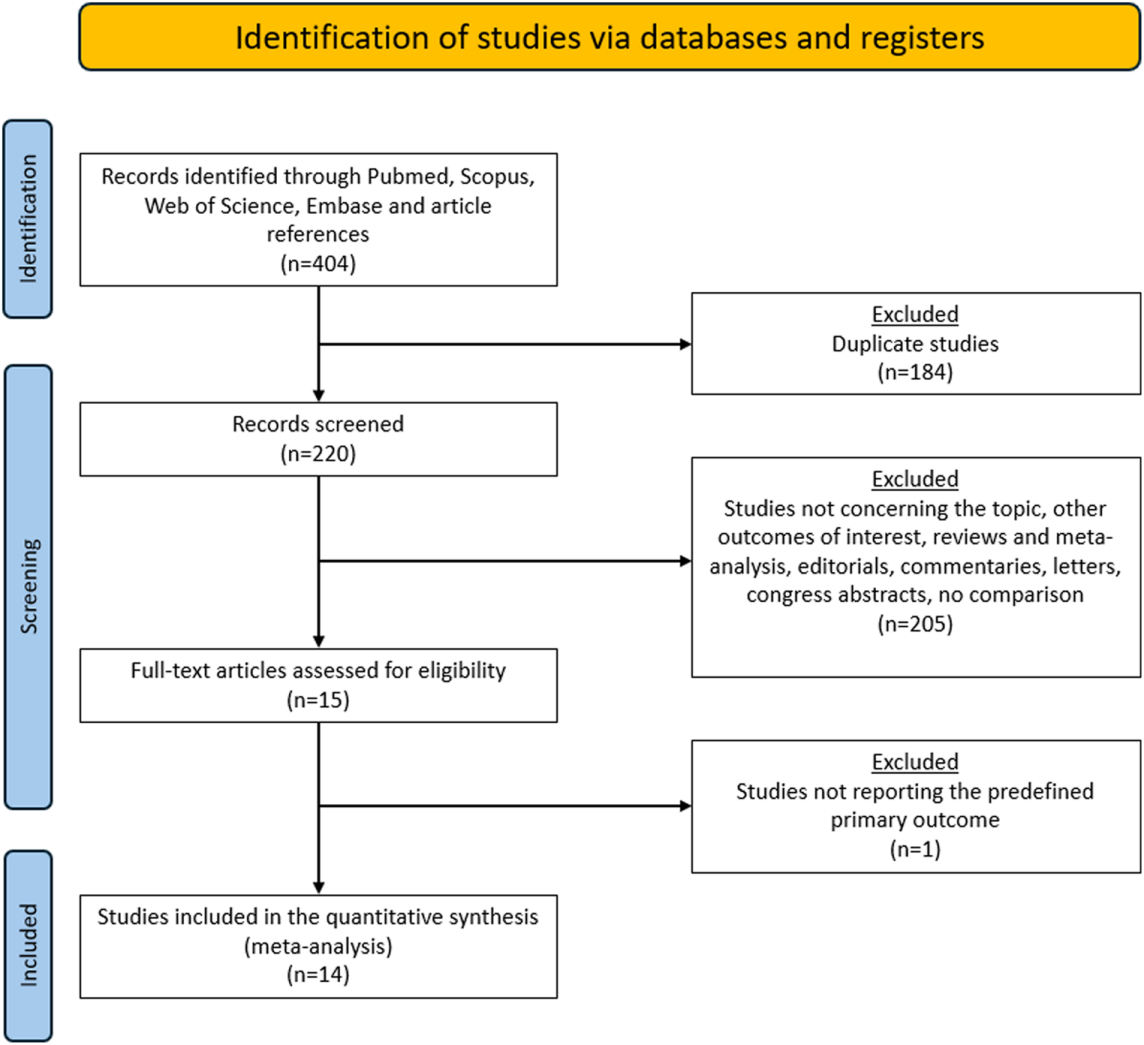
The preferred reporting items for systematic reviews and meta-analyses (PRISMA) diagram

The PRISMA flow chart is reported in Fig. 1. A total of 404 publications were identified. After duplicates were excluded, 220 titles were screened, and abstracts were reviewed. After full text evaluation of 15 articles, 14 studies published between 2005 and 2023 met the inclusion/exclusion criteria and were comprised in the quantitative synthesis. All studies were observational while the specific quality of each study is depicted in Table 1 [15–17, 19–25, 38–41]. 

**Table 1:** Demographic, clinical, and operative data for patients undergoing RHM and LHM

Author, year, country, study design	No. pts	Surgical procedure & No. pts		Age (yrs)	Gender (M)	BMI (kg/m2)	ASA *≥* 3	Classification			Previous endoscopic treatment				Fundoplicatio				Tenchique for myotomy			Myotomy lenght (cm)		Risk of Bias*
								Type I	Type II	Type III	BTX	PD	BTX + PD	POEM	D	T	N	None	Mono	Bipo	Blunt	Total	Gastric	
Horgan et al., 2005, USA & Argentina, Ret [23]	121	LHM	62	48 ± 19	29	nr	nr	nr			1	16	0	0	62			0	●			8–9	2–3	Serious
		RHM	59	42 ± 19	29			nr			4	17	5	0	59			0	●			8–9	2–3	
Huffmanm et al. 2007, USA, Pros [20]	61	LHM	37	nr	23	nr	nr	nr			nr				2	32	0	3			●	5–7	0.5-1	Serious
		RHM	24		10			nr			nr				16	6	0	2	●			5–7	0.5-1	
Sanchez et al. 2012, Venezuela, Pros [38]	31	LHM	18	40.7	nr	nr	nr	nr			0	1	0	0	18	0	0	0			●	8	2	Serious
		RHM	13	38				nr			0	0	0	0	13	0	0	0			●	8	2	
Perry et al. 2014, USA, Ret [39]	75	LHM	19	47.8 ± 14	11	25.7 ± 5	3	nr			nr				4	13	0	2	●			8	> 2	Moderate
		RHM	56	47.5 ± 16	28	26.3 ± 6	18	nr			nr				1	55	0	0	●			8–10	> 2	
Kim et al. 2019, USA, Ret [40]	72	LHM	35	59.5	20	nr	nr	nr			11	7	0	0	27	0	0	8	nr			8.2	2.7	Serious
		RHM	37	61.7	16			nr			17	8	0	0	29	3	0	5				8.8	2.9	
Alì et al. 2020, USA, Ret [17]	84	LHM	40	55	22	nr	nr	nr			nr				40		0	0	nr			nr	nr	Moderate
		RHM	44	57.5	28			nr			nr				44		0	0				nr	nr	
Arcerito et al. 2022, USA, Ret [15]	111	LHM	96	nr	nr	nr	nr	nr			0	0	19	0	96	0	0	0	●			7–8	2-2.5	Moderate
		RHM	15					nr			0	0	2	0	15	0	0	0	●			7–8	2-2.5	
Chacko et al. 2022, USA, Ret [41]	11,562	LHM	9703	nr	nr	nr	nr	nr			nr				nr				nr			nr		Serious
		RHM	1859																					
Engwall-Gill et al. 2022, USA, Ret [19]	59	LHM	14	43	9	nr	nr	nr			4	0	0	0	7	0	0	7	●			8	2	Moderate
		RHM	45	54	17			nr			4	0	0	0	11	0	0	34	●			8	2	
Gass et al. 2022, Switzerland, Pros [24]	43	LHM	32	54.9	23	23.7	4	10	15	4	0	6	0	0	32	0	0	0	●			> 10	nr	Moderate
		RHM	11	60.5	5	23.1	5	2	9	0	0	1	0	0	11	0	0	0	●			> 10	nr	
Raja et al. 2022, USA, Ret PM [22]	447	LHM	277	49 ± 16	141	26 ± 5	159	59	170	12	36	26	0	0	277	0	0	0		●		> 7	> 3	Moderate
		RHM	170	50 ± 16	92	26 ± 5	134	22	116	3	13	2	0	0	170	0	0	0		●		> 7	> 3	
Ilie et al. 2023, Romania, Ret [25]	152	LHM	69	51.2	38	nr	nr	nr			0	0	0	0	36	2	0	31	nr			nr	nr	Moderate
		RHM	83	47.1	40			nr			0	0	0	0	64	2	3	14				nr	nr	
Jiang et al. 2023, China, Ret [16]	66	LHM	26	44.8 ± 11.4	14	20.8 ± 3	nr	7	14	2	0	7	0	0	26	0	0	0	●			5–6	2	Moderate
		RHM	40	43.1 ± 18.9	18	22.1 ± 3		10	22	3	0	7	0	2	40	0	0	0	●			5–6	2	
Rabe et al. 2023, Germany, Ret [21]	78	LHM	31	43.5	13	24	nr	16	13	2	3	19	0	6	31	0	0	0			●	7–9	2–3	Moderate
		RHM	47	53	25	24		16	30	1	2	15	0	0	47	0	0	0			●	7–9	2–3	

Ret retrospective; pros prospective; PM propensity score matching; pts patients; yrs years; M male sex; BMI body mass index; ASA American Society of Anesthesiology classification; BTX botulin toxin injection; PD pneumatic dilation; POEM peroral endoscopic myotomy; D Dor; T toupet; N Nissen; Mono monopolar energy device; Bipo bipolar energy device; Blunt blunt dissection with no utilization of electrocautery device. Data are reported as numbers, mean ± standard deviation, and median (range). Nr not reported. * the quality of included studies was assessed with the ROBINS-I tool for observational studies

Overall, 12,962 patients were included (Table 1). Of those, 2503 (19.3%) patients underwent RHM. The patient age ranged from 34 to 66 years, 51.7% were males, and the preoperative BMI ranged from 20.8 to 31 kg/m^2^. The specific achalasia subtype according to the Chicago Classification system was detailed in four studies: type I (25.5%), type II (69.7%), and type III (4.8%) [16, 21, 22, 24]. Only two studies detailed and reported and included data for patients diagnosed with stage III or stage IV disease according to the radiological classification [15, 22]. Ten studies detailed previous endoscopic intervention in 22.1% of patients that underwent botulin toxin injection (36.4%) [19, 21–23, 40], pneumatic dilation (50.6%) [16, 21–24, 38, 40], both (9.9%) [15, 23], and peroral endoscopic myotomy (POEM) (3.1%)[16, 21]. Overall, 62.1% of previous endoscopic procedures were performed in patients undergoing LHM. In total, 11 studies reported on the length of esophageal and gastric myotomy (Table 1) [15, 16, 19–24, 38–40]. Among these, 3 studies detailed the use of intraoperative endoscopy to assess the myotomy length, while only one study mentioned the use of intraoperative manometry to assess the resolution of the LES high-pressure zone [16, 20, 24]. None of the studies included in the analysis discussed the use of EndoFLIP technology. Additionally, three studies addressed the integrity of the mucosa after myotomy through intraoperative endoscopy with hydropneumatic bubbling test (2 studies) or dye blue methylene test (1 study) [15].

Overall, 10 studies described the surgical technique for esophageal myotomy detailing the utilization, for both LHM and RHM, of monopolar energy devices (6 studies) [15, 16, 19, 23, 24, 39], bipolar energy device (1 study) [22], and blunt dissection (2 studies) [21, 38]. Huffmann et al. [20] described blunt dissection in LHM and monopolar dissection for RHM patients. None of the studies described the power of energy device (wattage) or utilization of ultrasound or radiofrequency devices for LHM. EP was diagnosed in 259 patients; however, none of the studies provided specific data on defect size. Eleven studies reported the timing of diagnosis for 58 EP, with 96.5% being diagnosed intraoperatively [15, 17, 19–24, 38–40]. In cases of intraoperative diagnosis, the EP was closed using interrupted absorbable sutures and reinforced with the anterior fundoplication. Fundoplication after myotomy was detailed in 13 studies; [15–17, 19–25, 38–40] Dor fundoplication was performed in 81.4% of patients, followed by Toupet fundoplication (9.4%), no fundoplication (8.8%), and Nissen fundoplication (0.4%). The type of robotic platform was specified in eight studies [15, 16, 20–24, 38] reporting the experience with the DaVinci S, DaVinci Si, and DaVinci Xi technologies. None of the included studies detailed the individual operating surgeon proficiency for both the laparoscopic and robotic approach.

Postoperative follow-up varied from 1 to 120 months, showing a trend of longer follow-up durations in the LHM group. Postoperative patients’ quality of life was detailed heterogeneously in seven studies [16, 20, 23, 24, 38, 39] with questionnaires (SF-36, GERD-HRQL, GIQLI) and institutional satisfaction scales (**Supplementary Table 1**).

### Meta-analysis- esophageal perforation (EP)

**Fig. 2:**
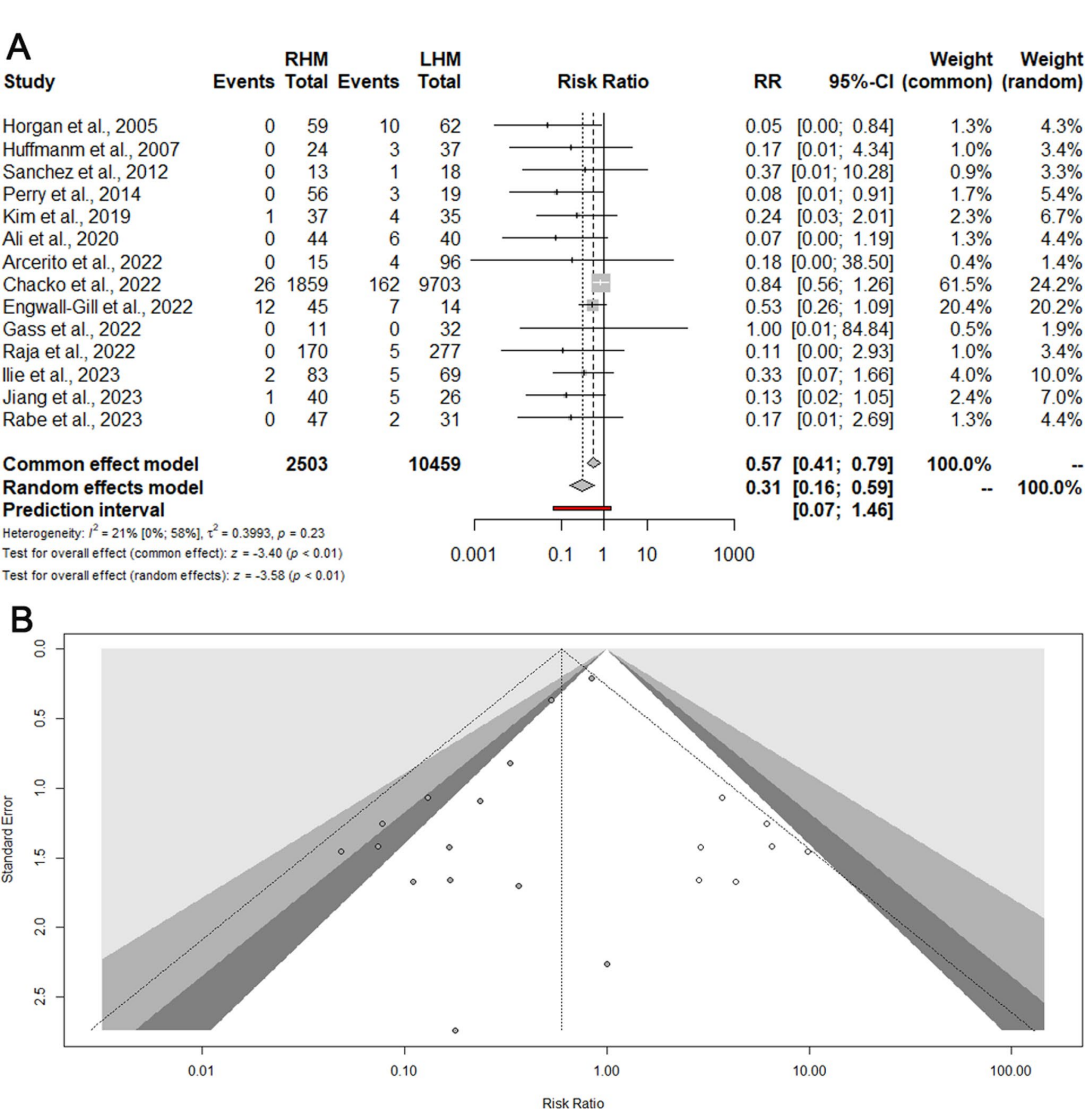
Esophageal perforation. Forrest (**A**) and Funnel (**B**) plot. RR: Risk ratio; 95% CI: Confidence Interval

EP was reported in 14 studies (12962 patients) [15–17, 19–25, 38–41]. The cumulative incidence of EP was 1.67% for RHM and 2.07% for LHM. Compared to LHM, RHM was associated to a significantly reduced EP risk (RR: 0.31; 95% CI 0.16–0.59) (Fig. 2A). The prediction lower and upper limits were 0.07 and 1.46, respectively. The heterogeneity was low (I^2^ = 21%) and τ^2^ = 0.39. The Funnel plot (Fig. 2B) and the Egger test did not show evidence of publication bias. The sensitivity analysis showed that omitting the study by Chacko et al. [41] the heterogeneity decreased to 0.0% with a preserved statistically significant risk reduction for RHM (RR: 0.25; 95% CI 0.13–0.48).

### Secondary outcomes

Operative time, intraoperative blood loss, HLOS, and conversion rates to open were comparable between RHM vs. LHM (Table 2).

**Table 2:** Summary of the analysis of the categorical and continuous outcomes comparing RHM vs. LHM

Outcomes	No. Studies	No. pts		Heterogeneity	
				I^2^ (%)	95% CI
Dysphagia requiring reintervention or endoscopic procedures [15, 16, 22, 23, 25, 39, 40]	7	1044	0.47 (0.20–1.09) ^♦^	54	0–80
Conversion to open [15–17, 20, 21, 23–25, 38, 40]	11	894	0.36 (0.13–1.05) ^♦^	0.0	0–60
Post operative Eckardt score [16, 21, 25, 40]	4	368	-0.42 (-0.94, 0.11) ^*^	83	57–93
Estimated intraoperative blood loss (ml) [16, 20, 23, 39, 40]	5	395	-0.46 (-0.98, 0.08) ^*^	80	54–92
Operative time (min) [15–17, 20–25, 38–40]	12	1341	-0.34 (-1.90, 1.21) ^*^	98	97–99
HLOS (day) [17, 19, 20–25, 38–41]	12	12,785	-0.25 (-0.43, 0.06) ^*^	63	31–80

♦ Risk ratio, * Standardized mean difference, (95% Confidence Interval), I [2] Heterogeneity

### Functional outcomes

**Fig. 3:**
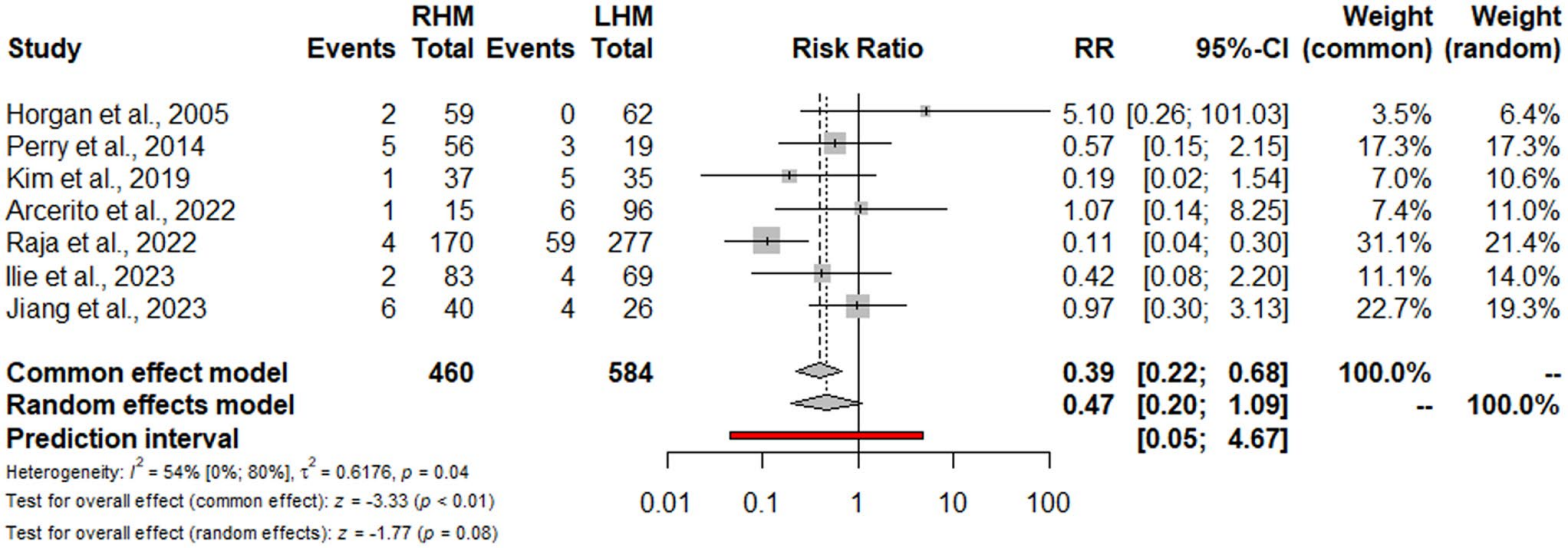
Dysphagia requiring reoperation or additional endoscopic procedures. Forrest plot. RR: Risk ratio; 95% CI: Confidence Interval

**Fig. 4:**
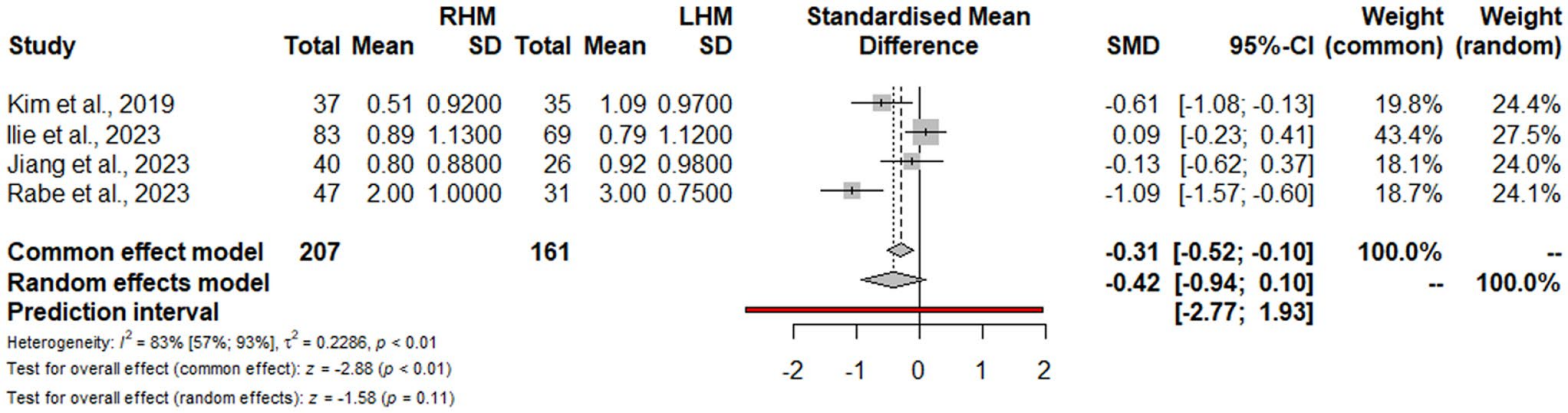
Postoperative Eckardt score. Forrest plot. SMD: standardized mean difference; 95% CI: Confidence Interval

Dysphagia requiring reoperation or additional endoscopic procedures was detailed in 7 studies (1044 patients) [15, 16, 22, 23, 25, 39, 40], with no significant differences for RHM vs. LHM (RR: 0.47; 95% CI 0.20–1.09; I^2^ = 54%) (Fig. 3). The sensitivity analysis showed that omitting the study by Raja et al. [22] the heterogeneity decreases to 0.0% with a preserved nonsignificant result (RR: 0.69; 95% CI 0.35–1.34). The postoperative Eckardt score was reported in four studies (368 patients) [16, 21, 25, 40]. No significant differences were found for RHM vs. LHM (SMD: -0.42; 95% CI -0.94, 0.11; I^2^ = 83%) (Fig. 4). The sensitivity analysis showed the robustness of findings in term of point estimation, 95% CI, and heterogeneity. The Funnel plot (Fig. 2B) and the Egger test did not show evidence of publication bias for both functional outcomes. Using the GRADE tool, we rated the quality of evidence for the primary and secondary outcomes supporting each outcome as moderate mostly due to the limitations in study design (**Supplementary Table 2**).

## Discussion

This meta-analysis shows that the overall risk of EP related to surgical myotomy for the treatment of achalasia is about 2%. RHM may be associated with a reduced risk of EP compared to LHM however, caution is mandatory because the hidden impact of several confounders such as outcome reporting (detection bias), surgeon expertise, impact of prior endoscopic procedures at the LES, and variations of surgical techniques (i.e., type of energy devices, wattage, type of dissection, type of fundoplication etc.). Both LHM and RHM have comparable functional effect on postoperative dysphagia requiring reoperation or additional endoscopic procedures and decrease of postoperative Eckard score.

Minimally invasive Heller myotomy is the preferred surgical option for treating symptomatic achalasia [4, 8, 9, 42–45]. Numerous studies indicate that LHM is both safe and effective in the medium to long term follow-up, achieving symptom relief in 80–100% of patients after five years and 75% after 15 years [8, 9, 42, 46]. The risk of complications with LHM is approximately 6.3%, while the mortality risk is just 0.1% [8, 47–51]. Since its introduction in 2001, RHM has gained widespread acceptance worldwide due to its improved three-dimensional visualization, greater range of motion of robotic arms, and enhanced precision during the procedure, potentially lowering the risk of iatrogenic full-thickness perforations [11, 20, 23]. EP during myotomy can occur in 1–3% of patients and may arise from direct injury to the esophageal mucosa during blunt dissection or from thermal damage due to electrocautery or energy devices. Thermal injury may lead to immediate or late perforation caused by fall of eschar at the myotomy site. When promptly identified intraoperatively, the defect should be sutured with interrupted stitches and patched with an anterior fundoplication [19, 49]. Esophageal myotomy remains challenging because of the difficult identification of the correct mucosal plane particularly in patients with esophageal dilation and mucosal redundancy and the possible presence of adhesions between the mucosal and the inner circular muscular layer in patients with previous endoscopic treatments [19, 47, 52]. For these reasons, preoperative esophageal lavage with removal of food debris, surgeon expertise, and cautious surgical dissection can minimize the risk of EP [50]. In our study, the pooled cumulative risk of EP for RHM and LHM was 1.67% and 2.07%, respectively, consistent with literature data. The quantitative analysis suggested a significantly reduced EP risk for RHM vs. LHM (RR: 0.31; 95% CI 0.16–0.59). This result was also supported by the sensitivity 0% heterogeneity analysis (RR: 0.25; 95% CI 0.13–0.48). A previous meta-analysis by Milone et al. including 6 studies (2625 patients), showed a significantly reduced odds for EP in RHM (OR: 0.13; *p* < 0.001) [14]. Similarly, Ataya et al. reported a significantly reduced odds for EP in patients undergoing RHM compared to LHM (OR: 0.36; *p* = 0.02) [18]. Also, Raja et al. in their retrospective single center analysis of 447 patients reported a lower EP rate for RHM vs. LHM (0% vs. 1.8%) [22]. These results highlight the benefits of robotic surgery that offer a wide range of motion, motion scaling with tremor filtration, high-definition magnification, binocular three-dimensional visualization of the surgical area, and enhanced ergonomic comfort for the surgeon. These features of the robot-assisted system likely contribute to improved clinical outcomes by facilitating safer dissections, enhancing visualization, and increasing instrument maneuverability. In the setting of the Heller myotomy, the advanced 3D imaging and the stability of the surgical field allows a more precise identification of both longitudinal and circular esophageal muscle fibers. Furthermore, the magnification helps the surgeon to better identify the submucosal plane and to safeguard against perforation [11–14]. Despite the low or 0% heterogeneity, several factors should be considered when interpreting our results. First, the proficiency of the individual operating surgeons was not reported in any of the included studies. Second, the techniques used for esophageal myotomy were inconsistently documented, with none of the studies mentioning the use of ultrasound or radiofrequency instruments and details such as the wattage of the monopolar devices. No specific details were reported about the technique of blunt dissection in the body of the esophagus and at the esophagogastric junction. Third, although RHM is a newer approach compared to LHM, it is possible that confounding bias exists, as RHM is often performed by skilled minimally invasive surgeons who have already mastered the LHM technique. Fourth, data on the degree of esophageal dilation was limited and heterogeneous across the included studies, with only two of these providing details on stage III-IV patients, who present greater challenges due to mucosal redundancy. Fifth, a higher proportion of patients undergoing LHM had a history of previous endoscopic treatment, which may be associated with submucosal-mucosal adhesions, complicating surgical dissection and increasing the risk of mucosal violation [52]. Finally, 96.5% EP were identified during surgery, promptly sutured and patched, thus limiting the postoperative clinical significance of such events with no impact on postoperative HLOS. Future studies should likely focus more closely on delayed perforations, which have a greater impact on clinical outcomes, overall morbidity, and longer length of stay.

Persistent or recurrent dysphagia after Heller myotomy is observed in up to 10% of patients and may necessitate revisional procedures such as endoscopic dilation, POEM, or redo myotomy [52–57]. Dysphagia can result from incomplete myotomy, tissue fibrosis and scarring, peptic stricture, diverticulum at the myotomy site, twisted fundoplication, hiatal hernia, progressive esophageal dilation leading to a sigmoid shape esophagus, or long-term cancer development [53–55, 58–60]. Importantly, our study found no significant differences in functional outcomes regarding dysphagia necessitating additional endoscopic interventions or reoperations and the postoperative Eckardt score. This aligns with findings from Milone et al. and Ataya et al., which reported similar functional outcomes for LHM and RHM [14, 18]. This may be attributed to the fact that functional outcomes following myotomy depend more on factors such as the length of myotomy, preoperative manometric classification, degree of esophageal dilation, the surgeon’s experience, and follow-up, rather than the surgical approach itself [9, 14, 18, 24, 25, 61]. In our analysis the follow-up duration ranged from 1 to 120 months. Notably, the follow-up was generally longer in the LHM group compared to the RHM group, which may have introduced a temporal bias in the recorded quality of life and satisfaction outcomes. Finally, no notable differences were observed between treatment methods concerning operating time, HLOS, intraoperative blood loss, or conversion to open surgery. In terms of postoperative quality of life, data were inconsistently reported across different follow-up time points, making robust quantitative analysis difficult. However, Huffmann et al. [20]. noted a significantly higher quality of life postoperatively for RHM compared to LHM, while other studies reported no differences [20]. Future research focusing on quality of life and patient-reported outcomes is needed to further explore this important aspect of the procedure.

We recognize that our paper has several limitations, primarily related to the retrospective designs of the included studies, which may introduce preoperative selection and reporting biases. In the quantitative analysis, the study by Chacko et al. [41] included a substantial number of patients, which may have impacted the overall effect globally. Nevertheless, the use of random effects analysis in the quantitative assessment moderated the influence of this large study. Additionally, only a few studies provided information on the operating surgeon’s proficiency, which could significantly affect myotomy-related outcomes. Furthermore, there was a lack of detailed technical information about the surgical methodology for myotomy (such as wattage and whether a bottom-up or up-down technique was used), with none of the studies reporting on myotomy performed with an ultrasonic scalpel. The functional outcomes and quality of life measures were reported inconsistently across different follow-up time points. Importantly, even when studies reported on patients’ symptoms, it remains challenging to assess therapeutic success based solely on postoperative symptoms, due to potential exaggeration of symptom improvement and altered swallowing perceptions. The absence of objective postoperative assessments in most studies (i.e., esophageal emptying) might contribute to the potential discordance between esophageal emptying and symptoms. Lastly, related costs were heterogeneously reported in few studies thus impeding a robust quantitative assessment.

## Conclusions

Our analysis suggests that RHM may be linked to a lower risk of EP compared to LHM. However, because of preoperative selection bias, variations in surgeon expertise, prior endoscopic procedures, and the techniques employed for the myotomy, the current findings should not be viewed as conclusive, and the superiority of one approach over the other remains to be established.

## Electronic supplementary material

Below is the link to the electronic supplementary material.

Supplementary Material 1

Supplementary Material 2

## Data Availability

No datasets were generated or analysed during the current study.
