# Supplementary material for: Robotic versus laparoscopic heller myotomy for esophageal achalasia: an updated systematic review and meta-analysis
Source: Langenbecks Arch Surg. 2025 Feb 17;410(1):75. doi: 10.1007/s00423-025-03648-1 (PMC11832576; doi:10.1007/s00423-025-03648-1)
Supplement: Supplementary file 1 — Supplementary Material 1 [file 423_2025_3648_MOESM1_ESM.docx]

| **Author** | **Surgical procedure** | **Quality of life assessment** | **Mean follow-up (mos)** |  |
| --- | --- | --- | --- | --- |
|  |  |  |  |  |
| Horgan et al.^23^ | LHM | 90% of patients consider their swallowing status as good/excellent | 18 |  |
|  | RHM | 92% of patients consider their swallowing status as good/excellent | 18 |  |
| Huffmanm et al.^20^ | LHM | SF-36 (Role functioning, emotional): 87 SF-36 (General Health Perception): 68 | 1-6^*^ |  |
|  | RHM | SF-36 (Role functioning, emotional): 74 SF-36 (General Health Perception): 53 | 1-6^*^ |  |
| Sanchez et al.^38^ | LHM | Symptom relief: 94.5% | 18 |  |
|  | RHM | Symptom relief: 100% | 18 |  |
| Perry et al.^39^ | LHM | GERD-HRQL: 12 (6-20) Satisfied 91% | 120 |  |
|  | RHM | GERD-HRQL: 11 (0-36) Satisfied 95.5% | 108 |  |
| Kim et al.^40^ | LHM | nr | nr |  |
|  | RHM |  |  |  |
| Alì et al.^17^ | LHM | nr | nr |  |
|  | RHM |  |  |  |
| Arcerito et al.^15^ | LHM | nr | nr |  |
|  | RHM |  |  |  |
| Chacko et. al.^41^ | LHM | nr | nr |  |
|  | RHM |  |  |  |
| Engwall-Gill et al.^19^ | LHM | nr | nr |  |
|  | RHM |  |  |  |
| Gass et al.^24^ | LHM | GERD-HRQL: 4 (2-10) Satisfied 87.5% | 89 |  |
|  | RHM | GERD-HRQL: 4 (2-9) Satisfied 66.7% | 28 |  |
| Raja et al.^22^ | LHM | nr | nr |  |
|  | RHM |  |  |  |
| Ilie et al.^25^ | LHM | nr | nr |  |
|  | RHM |  |  |  |
| Jiang et al.^16^ | LHM | STOOL 2.2 ± 0.7 GIQLI 117.7 ± 7.1 | 57 |  |
|  | RHM | STOOL 0.13 ± 0.4 GIQLI 120.8 ± 8.4 | 35 |  |
| Rabe et al.^21^ | LHM | nr | nr |  |
|  | RHM |  |  |  |
