# Supplementary material for: Robotic versus laparoscopic heller myotomy for esophageal achalasia: an updated systematic review and meta-analysis
Source: Langenbecks Arch Surg. 2025 Feb 17;410(1):75. doi: 10.1007/s00423-025-03648-1 (PMC11832576; doi:10.1007/s00423-025-03648-1)
Supplement: Supplementary file 2 — Supplementary Material 2 [file 423_2025_3648_MOESM2_ESM.pdf]

Certainty assessment							№ of patients		Effect		Certainty	Importance
№ of studies	Study design	Risk of bias	Inconsistency	Indirectness	Imprecision	Other considerations	RHM	LHM	Relative (95% CI)	Absolute (95% CI)		
**Esophageal perforation**												
14	non-randomised studies	serious	not serious	not serious	serious	all plausible residual confounding would reduce the demonstrated effect	43/2503 (1.7%)	216/10459 (2.1%)	RR 0.31 (0.16 to 0.59)	14 fewer per 1.000 (from 17 fewer to 8 fewer)	⊕⊕⊕◯ Moderate	CRITICAL
**Dysphagia requiring reintervention or endoscopic procedures**												
7	non-randomised studies	serious	serious	not serious	serious	all plausible residual confounding would reduce the demonstrated effect	21/460 (4.6%)	81/584 (13.9%)	RR 0.47 (0.20 to 1.09)	74 fewer per 1.000 (from 111 fewer to 12 more)	⊕⊕◯◯ Low	CRITICAL
**Post operative Eckardt score**												
4	non-randomised studies	serious	serious	serious	serious	all plausible residual confounding would reduce the demonstrated effect	207	161	-	SMD 0.42 SD lower (0.94 lower to 0.11 higher)	⊕◯◯◯ Very low	CRITICAL
**Conversion to open**												
11	non-randomised studies	not serious	not serious	not serious	not serious	none	0/429 (0.0%)	10/465 (2.2%)	RR 0.36 (0.13 to 1.05)	14 fewer per 1.000 (from 19 fewer to 1 more)	⊕⊕⊕⊕ High	CRITICAL

Certainty assessment							№ of patients		Effect		Certainty	Importance
№ of studies	Study design	Risk of bias	Inconsistency	Indirectness	Imprecision	Other considerations	RHM	LHM	Relative (95% CI)	Absolute (95% CI)		
**Intraoperative blood loss**												
5	non-randomised studies	not serious	not serious	not serious	not serious	none	216	179	-	SMD 0.46 SD lower (0.98 lower to 0.08 higher)	⨁⨁⨁⨁ High	IMPORTANT
**Operative time**												
12	non-randomised studies	serious	serious	serious	serious	all plausible residual confounding would reduce the demonstrated effect dose response gradient	599	742	-	SMD 0.34 SD lower (1.9 lower to 1.21 higher)	⨁⨁◯◯ Low	CRITICAL
**HLOS**												
12	non-randomised studies	serious	serious	serious	serious	publication bias strongly suspected all plausible residual confounding would reduce the demonstrated effect	2448	10337	-	SMD 0.25 SD lower (0.43 lower to 0.06 higher)	⨁◯◯◯ Very low	CRITICAL

Supplementary Table 2. Grading of Recommendations, Assessment, Development, and Evaluation framework (GRADE) stratified for different study outcomes. CI: confidence interval; RR: risk ratio; SMD: standardised mean difference
